# The XCL1/XCR1 axis is upregulated in type 1 diabetes and aggravates its pathogenesis

**DOI:** 10.1172/jci.insight.178743

**Published:** 2025-02-27

**Authors:** Camilla Tondello, Christine Bender, Gregory J. Golden, Deborah Puppe, Elisa Blickberndt, Monika Bayer, Giulia K. Buchmann, Josef Pfeilschifter, Malte Bachmann, Edith Hintermann, Ralf P. Brandes, Michael R. Betts, Richard A. Kroczek, Urs Christen

**Affiliations:** 1 Institute for Pharmacology and Toxicology, Pharmazentrum Frankfurt/ZAFES, Goethe University Frankfurt, Frankfurt, Germany.; 2 Perelman School of Medicine University of Pennsylvania, Philadelphia, USA.; 3 Institute for Cardiovascular Physiology, Goethe University Frankfurt, Frankfurt, Germany.; 4 Deutsches Zentrum für Herz-Kreislauferkrankungen (DZHK), Partnersite RhineMain, Frankfurt, Germany.; 5 Robert Koch Institute, Berlin, Germany.

**Keywords:** Autoimmunity, Immunology, Chemokines, Dendritic cells, T cells

## Abstract

Type 1 diabetes (T1D) is precipitated by the autoimmune destruction of the insulin-producing β cells in the pancreatic islets of Langerhans. Chemokines have been identified as major conductors of islet infiltration by autoaggressive leukocytes, including antigen-presenting cells and islet autoantigen–specific T cells. We have previously generated a road map of gene expression in the islet microenvironment during T1D in a mouse model and found that most of the chemokine axes are chronically upregulated during T1D. The XCL1/XCR1 chemokine axis is of particular interest, since XCR1 is exclusively expressed on conventional DCs type 1 (cDC1) that excel by their high capacity for T cell activation. Here, we demonstrate that cDC1-expressing XCR1 are present in and around the islets of patients with T1D and of individuals with islet autoantibody positivity. Furthermore, we show that XCL1 plays an important role in the attraction of highly potent DCs expressing XCR1 to the islets in an inducible mouse model for T1D. XCL1-deficient mice display a diminished infiltration of XCR1^+^ cDC1 and, subsequently, a reduced magnitude and activity of islet autoantigen–specific T cells, resulting in a profound decrease in T1D incidence. Interference with the XCL1/XCR1 chemokine axis might constitute a novel therapy for T1D.

## Abstract 2

The chemokine axis XCL1/XCR1 is expressed in type 1 diabetes patients. A interference with this axis ameliorates type 1 diabetes in a mouse model.

## Graphical abstract

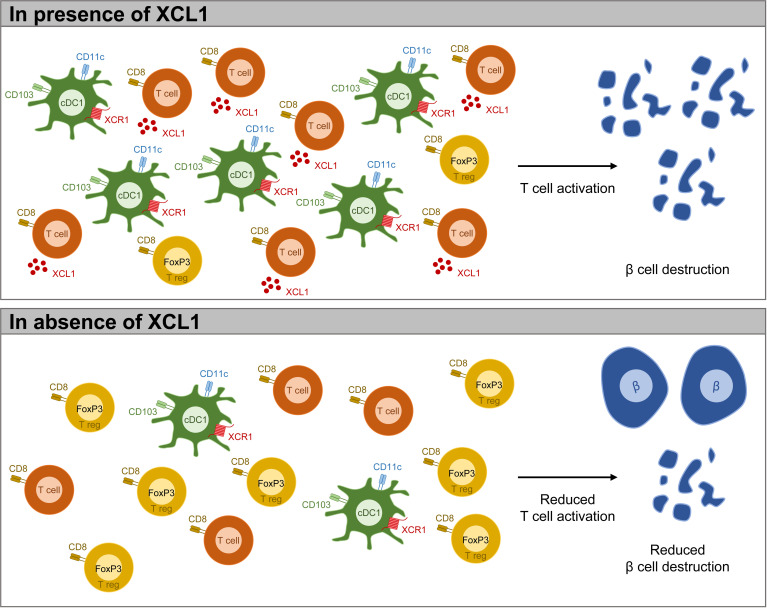

## Introduction

Type 1 diabetes (T1D) is a severe autoimmune disease affecting the insulin-producing β cells in the pancreatic islets of Langerhans. Currently, treatment of T1D is largely restricted to a symptomatic insulin-replacement therapy. Due to the effectiveness of insulin and the possible side effects of general immunosuppressive drugs, almost no therapies targeting the autoreactive immune system are being used in clinical practice. Nevertheless, many patients with T1D suffer from long-term adverse effects, such as diabetic nephropathy, cardiovascular diseases, retinopathy, neuropathy, and diabetic foot disease. In addition, patients with T1D live with the constant danger of encountering hypoglycemic events that, especially when occurring at night, pose a very serious threat to life. Fortunately, the anti-CD3 antibody teplizumab (1, 2) has recently been approved by the FDA for the treatment of patients with stage 2 T1D (3). However, the efficacy of teplizumab is only transient, and many patients do not respond to the treatment (2). Thus, the medical need for novel immunotherapies of T1D is immense.

The migration of aggressive T cells and other immune cells to the pancreas and the subsequent infiltration of the islets of Langerhans are orchestrated by chemokines that act as chemoattractants for cells carrying the respective chemokine receptor. We have recently performed a detailed mapping of the chemokine expression pattern in the islet microenvironment before and after T1D initiation (4). For this endeavor, we used the inducible RIP-LCMV-GP (RIP-GP) model in which the glycoprotein (GP) of the lymphocytic choriomeningitis virus (LCMV) is expressed in the β cells of the islets of Langerhans under control of the rat insulin promoter (RIP) (5). Infection of such RIP-GP mice with LCMV initiates an autoaggressive immune response that destroys β cells and results in a fast-onset T1D that manifests within 10–14 days after infection. We found that the expression of several chemokine axes including CXCL10/CXCR3, CCL5/CCR5, CXCL16/CXCR6, and XCL1/XCR1 was strongly regulated in a manner that persisted in the chronic phase of T1D (4). Whereas the critical role of the CXCL10/CXCR3 axis in attracting aggressive T cells to the islets, and thereby driving the immunopathogenesis of T1D, has been characterized extensively in patients and in animal models (6–10), the role of the XCL1/XCR1 axis is largely unknown.

Among the ligands and receptors of the chemokine network, the XCL1/XCR1 axis is unique, due to the fact that XCR1 is exclusively expressed on conventional DCs type 1 (cDC1) (11, 12). Such cDC1 display an aggressive phenotype characterized by the production of type I and II IFNs as well as a high cross-presentation activity. They are important in cellular immunity against tumor and intracellular pathogens by inducing a strong Th1 immune response. In the mouse, resident and migratory cDC1 can be further distinguished primarily due to the surface expression of CD8α and CD103, respectively, whereas both express XCR1, MHCII, and CD11c (12, 13). XCL1 is predominately released by T cells and NK cells. Thus, the XCL1/XCR1 axis is important for the crosstalk between cDC1 and T cells and, subsequently, for the effective activation of aggressive T cells (14). In contrast to mice, humans also express the XCL1 paralog XCL2. XCL1 and XCL2 are structurally highly homologous and have similar biological properties (15, 16). However, XCL2 seems to display slightly higher affinity to heparin (16), and the pattern of XCL1 and XCL2 expression is different in immune cell subpopulations, such as CD4 T cells, which predominantly express XCL2 (17).

Here, we demonstrate that XCR1^+^ cDC1 are indeed found in the pancreas of patients with T1D as well as in prediabetic individuals carrying autoantibodies against islet autoantigens. We then further characterized the expression of XCL1 and XCR1 by RNAScope and investigated the role of the XCL1/XCR1 axis in the pathogenesis of T1D by crossing RIP-GP mice to XCL1-deficient mice. Such RIP-GP × XCL1^–/–^ mice showed reduced infiltration of cDC1 into the islets and, subsequently, a diminished number and overall fitness of autoaggressive, islet autoantigen–specific T cells. Hence, the incidence and severity of T1D was massively reduced in the absence of XCL1. Our data indicate that the XCL1/XCR1 axis plays an important role in the immunopathogenesis of T1D and might be an attractive novel target for immune intervention.

## Results

### XCL1 and XCR1 are upregulated in the islets of Langerhans in RIP-GP mice upon infection and are present in pancreatic islets of NOD mice.

**Figure 1:**
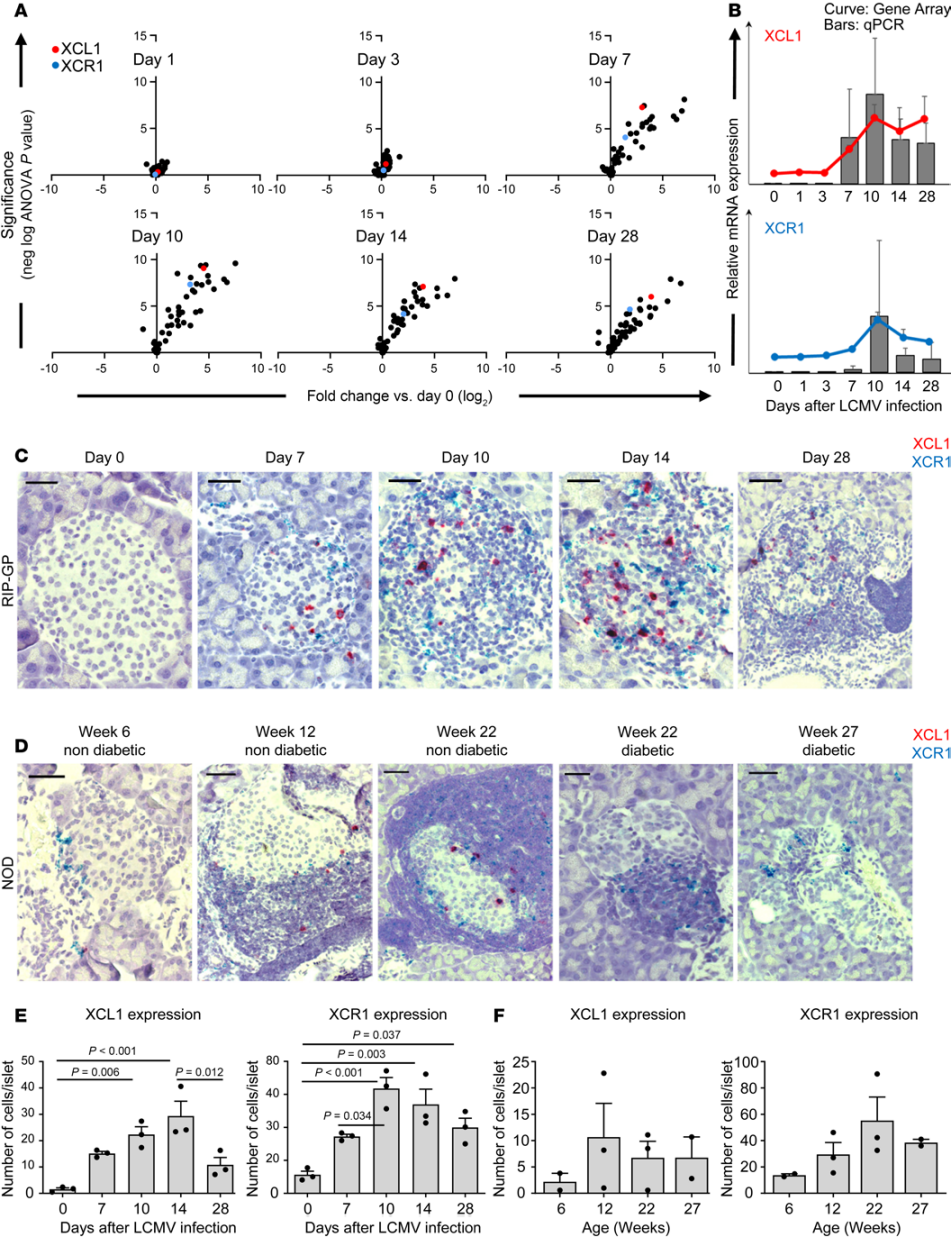
XCL1 and XCR1 are upregulated in the islets of RIP-GP mice and NOD mice. (**A**) Volcano blots of the gene expression profile of chemokine ligands and their receptors of laser-dissected islets from RIP-GP mice at day 1, 3, 7, 10, 14, and 28 after the LCMV infection in comparison with uninfected mice. XCL1 is highlighted in red and XCR1 in blue. (**B**) Quantification of the data obtained by gene array (curves) and reverse transcription PCR (RT-PCR) (bars) of laser-dissected islets for the expression of XCL1 and XCR1. Data are shown as mean ± SD. (**C**) Duplex RNAScope in situ hybridization XCL1 (red) and XCR1 (blue) of pancreas tissue sections from RIP-GP mice at days 0, 7, 10, 14, 28 after infection (*n* = 3 mice per time point). Representative images are displayed per time. Original magnification, 63× (oil). Scale bar: 20 μm. (**D**) Duplex RNAScope in situ hybridization for XCL1 (red) and XCR1 (blue) of pancreas tissue sections from NOD mice obtained at different age and disease state (*n* = 2–3). Mice were considered diabetic with nonfasting BG levels of > 300 mg/dL. Original magnification, 40×. Scale bars: 25 μm. (**E**) Quantification of XCL1 (left) and XCR1 (right) expression in the islets of RIP-GP mice at day 0, 7, 10, 14, and 28 after infection (*n* = 3 per time point). Data are shown as mean ± SEM, and significant *P* values (2-way ANOVA) are indicated. (**F**) Quantification of XCL1 (left) and XCR1 (right) expression in the islets of NOD mice at different time points (*n* = 2–3 per time point). Data are shown as mean ± SEM.

We previously performed laser dissection of the islet microenvironment of RIP-GP mice at several times after LCMV-infection followed by gene array analysis and quantitative PCR (qPCR) (4). Among many chemokine ligands and receptors that have been upregulated, a chemokine ligand/receptor pair turned out to be of particular interest due to its magnitude and persistence. Namely, XCL1 and its receptor XCR1 were strongly increased in expression starting at day 7 after infection, and importantly, they both remained upregulated through day 28 when the disease was already chronic. Here, we display these previous data again (4), focusing on the expression of XCL1 and XCR1 (Figure 1, A and B). To visualize and confirm that both XCL1 and XCR1 are indeed localized in the islet microenvironment of RIP-GP mice, we used RNAScope in situ hybridization to stain pancreas tissue sections for XCL1 and XCR1 (Figure 1C). At day 0, neither XCL1 nor XCR1 mRNA–producing cells were present in the islets. They started to appear at day 7 among the infiltrating cells and increased in numbers between days 10 and 14. At day 28, the presence of both the ligand and the receptor was decreased, but they were both still present in the islets. A quantification of the number of XCL1- and XCR1-expressing cells revealed a significant increase over time (Figure 1E). Importantly, even at day 28 after infection, during the chronic phase of the disease, both XCL1- and XCR1-expressing cells remain in the islets of Langerhans (Figure 1E). The kinetics of the increase in the number of XCL1- and XCR1-expressing cells in the islets is very similar to the kinetics observed for XCL1 and XCR1 RNA expression found by gene array and qPCR (Figure 1B).

To demonstrate that the XCL1/XCR1 axis is also expressed independently from a virus infection, we stained pancreas sections of NOD mice as well with the RNAScope duplex technique. Representative images of islets collected from mice at different ages and disease stage are shown (Figure 1D). At week 6 of age, these young NOD mice displayed only minor periinsulitis with only few cells expressing XCR1. At week 12, the mice were still in a nondiabetic state, but they started to show large periinsular clusters of infiltrating cells, with many of them expressing XCR1 or XCL1 and located in close proximity to each other. At an age of 22 weeks, NOD mice were grouped into diabetic and nondiabetic mice. The comparison between these 2 groups revealed that, in the islets of nondiabetic mice, many XCL1^+^ or XCR1^+^ cells remained present. In contrast, infiltrates in and around the islets of diabetic mice contained mainly cells expressing XCR1 but few expressing XCL1. The quantification of XCL1- and XCR1-expressing cells in the islets of NOD mice also followed a similar pattern as RIP-GP mice. However, due to the large heterogeneity between NOD mice of the same age, the tendency of increased numbers of XCL1- and XCR1-expressing cells was not significant (Figure 1F).

### XCR1^+^ cells are present in the pancreas of patients with T1D and in islet autoantibody–positive individuals.

**Figure 2:**
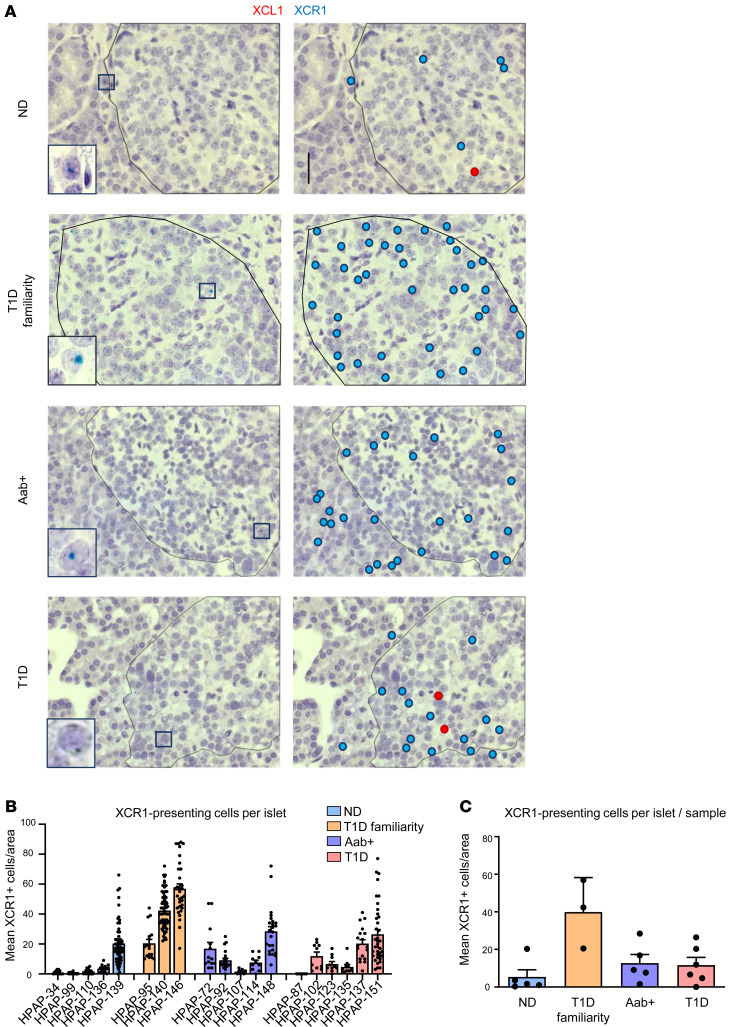
XCR1^+^ cDC1 are present in the islets of patients with T1D and individuals with islet autoantibodies. Human pancreas slides were obtained through the HPAP program. Groups of individuals were divided as follows: nondiabetic (ND), nondiabetic with known T1D familiarity (T1D familiarity), autoantibody positive (Aab^+^), and patients with diabetes (T1D). (**A**) Representative pictures of duplex RNAScope in situ hybridization for XCL1 (red) and XCR1 (blue). In the left column, the original picture with a magnified example of a positive cell (square). On the right, XCR1-expressing cells are highlighted with a light blue dot, and XCL1-expressing cells are highlighted with a red dot. Islets are indicated with black lines. Original magnification, 63×. Scale bars: 20 μm. (**B**) Number of XCR1-expressing cells per islet microenvironment. Number of islets per section analyzed was 6–63. Each islet is represented by 1 dot. Data are shown as the mean ± SEM number of XCR1-expressing cell in one individual. (**C**) Mean ± SEM of XCR1-expressing cells per islet microenvironment in the groups of ND, T1D familiarity, Aab^+^, and T1D. Number of islets per section analyzed was 6–63. The mean for every individual is represented by dots.

Sections of human pancreata from individuals at different disease stages obtained through the Human Pancreas Analysis Program (HPAP) were stained with RNAScope duplex technique for XCL1- and XCR1-expressing cells. Pancreata from nondiabetic donors (ND), ND with known T1D familiarity, autoantibody-positive donors (Aab^+^), and patients with diagnosed T1D were analyzed (Supplemental Table 1; supplemental material available online with this article; https://doi.org/10.1172/jci.insight.178743DS1). Tissue sections from Aab^+^ and T1D individuals contained more cells producing XCR1 mRNA compared with the pancreas of healthy controls (Figure 2A). XCR1^+^ cells were mostly found in or around the islets. XCR1-expressing cells were quantified by counting the positive cells in and around the islets. Even though not significant due to the high variation between individual islets, in both Aab^+^ and T1D pancreata, more XCR1^+^ cells per islet are present than in islets of tissue section of ND controls. Interestingly, the group with most XCR1^+^ cells was the individuals without diabetes (nondiabetic) with known T1D familiarity (Figure 2, B and C). Surprisingly, very few XCL1^+^ cells were present in any of the pancreas section, independently from the disease stage. However, this might be due to the low number of CD8 T cells present and/or by the transient chemokine RNA expression. Therefore, the timing of the staining might be crucial to visualize XCL1 expression. In addition, the RNA degradation in the collected tissue samples might also influence the staining for low-abundance RNA species.

### XCL1-deficient mice showed reduced numbers of cDC1 in the islet microenvironment.

**Figure 3:**
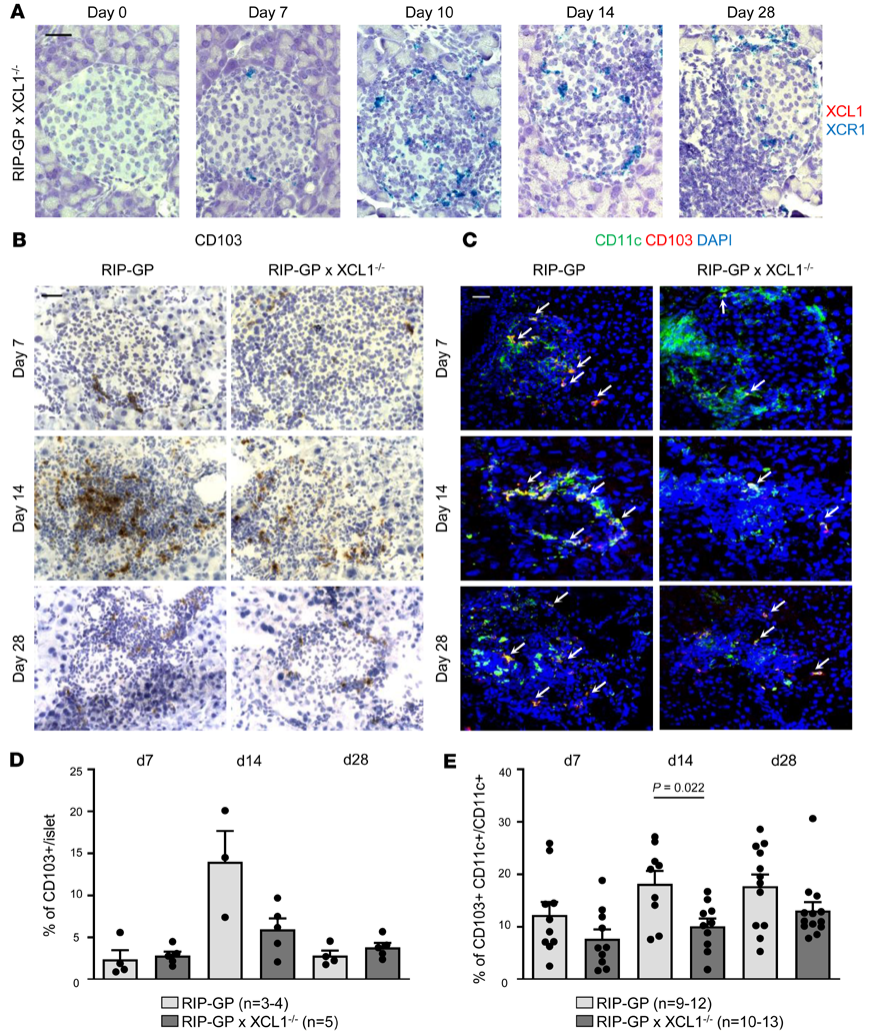
Fewer CD103^+^ cells are present in islets of XCL1-deficient mice. (**A**) Duplex RNAScope in situ hybridization for XCL1 (red) and XCR1 (blue) of pancreas tissue sections obtained from RIP-GP × XCL1^–/–^ mice at different times after infection, demonstrating that XCL1-deficient mice do not express XCL1. Original magnification, 63× (oil). Scale bars: 20 μm. (**B**) IHC staining for CD103 of quick-frozen pancreas sections at different times after LCMV infection, comparing RIP-GP with RIP-GP × XCL1^–/–^ mice. Representative pictures are shown. Original magnification, 40×. Scale bars: 25 μm. (**C**) Immunofluorescence double-staining for CD11c (green) and CD103 (red) of quick-frozen pancreas sections obtained at different times after LCMV infection, comparing RIP-GP with RIP-GP × XCL1^–/–^ mice. Nuclei are stained with DAPI (blue). White arrows indicate the double-positive cells (cDC1). Representative pictures are shown. Original magnification, 40×. Scale bars: 20 μm. (**D**) Quantification of the CD103 staining in RIP-GP (light gray) and RIP-GP × XCL1^–/–^ (dark gray) mice shown in **B**, expressed as a percentage of positive CD103 cell area per islet area. Numbers of mice per group are indicated. Data are shown as mean ± SEM. (**E**) Quantification of the CD103/CD11c double-staining in RIP-GP (light gray) and RIP-GP × XCL1^–/–^ (dark gray) mice shown in **C**, expressed as a percentage of CD103/CD11c–double-positive cell area per CD11c^+^ cell area. Numbers of mice per group are indicated. Data are shown as mean ± SEM, and significant *P* values (Mann-Whitney *t* test) are indicated.

To investigate whether the XCL1/XCR1 axis indeed plays a role in the pathogenesis of T1D, we generated XCL1-deficient RIP-GP mice by crossing RIP-GP mice with XCL1^–/–^ mice (11). These RIP-GP × XCL1^–/–^ mice were not generally immunosuppressed since the elimination of LCMV after infection was not altered in XCL1-deficient RIP-GP mice (Supplemental Figure 1). These data also confirm that the RIP-GP model is not influenced by an aberrant virus elimination in XCL1^–/–^ mice. XCL1-deficiency in RIP-GP × XCL1^–/–^ mice was confirmed by in situ hybridization with RNAScope duplex (Figure 3A). XCL1 was not expressed in XCL1-deficient RIP-GP mice before and after LCMV infection. In contrast, cells expressing XCR1 are also present in the islets of RIP-GP × XCL1^–/–^ mice, starting from day 7 after infection (Figure 3A). This indicates that, even in the absence of XCL1, many XCR1^+^ infiltrating cells are still attracted to the islets. It has been demonstrated that XCR1 is expressed exclusively on migratory cDC1 that, in addition, express CD103 (18). Thus, we assessed pancreas sections obtained from RIP-GP and RIP-GP × XCL1^–/–^ mice at days 7, 14, and 28 after LCMV infection for the presence of CD103^+^ cells (Figure 3, B–E). Indeed, at day 14 after infection, less CD103^+^ cells were found in RIP-GP × XCL1^–/–^ than in RIP-GP mice (Figure 3, B and D). However, since CD103 can also be found on some T cell subtypes, we performed double immunofluorescence staining for CD103 and the pan-DC marker CD11c and quantified the CD103^+^CD11c^+^ cells per CD11c^+^ cells (Figure 3, C and E). CD103^+^CD11c^+^ cells tended to be decreased in RIP-GP × XCL1^–/–^ mice at all times. In particular, at day 14 after infection, their frequency was reduced by 44% in RIP-GP × XCL1^–/–^ mice (*P* = 0.022).

**Figure 4:**
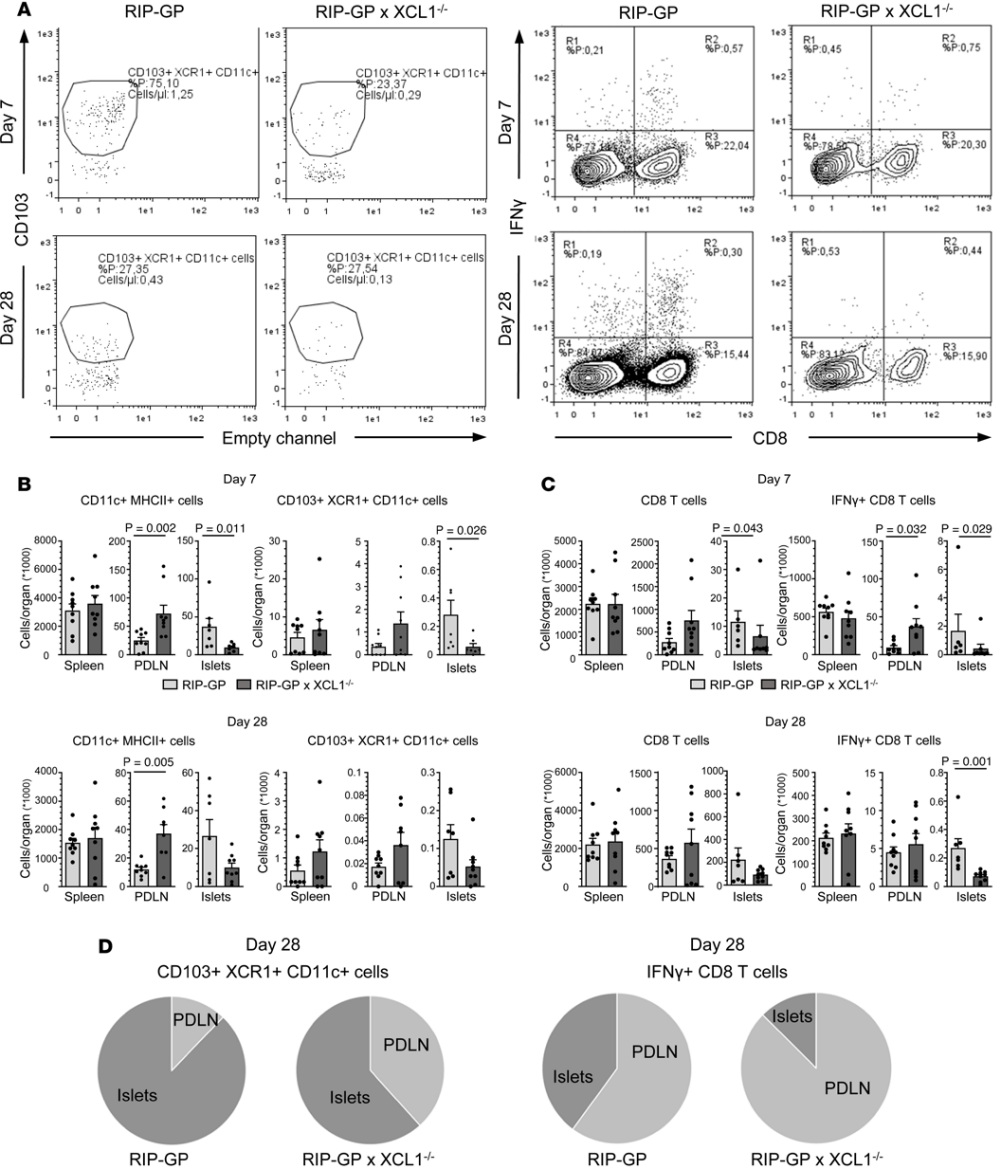
Numbers of DC and T cells are reduced in the islets but accumulate in the lymph nodes of XCL1-deficient mice. Quantification of different cell subtypes per organ (spleen, pancreatic draining lymph nodes, and islets) obtained via flow cytometric analysis at day 7 and day 28 after infection, comparing RIP-GP mice with RIP-GP × XCL1^–/–^ mice. (**A**) Representative dot plots of the most substantial changes in the islet-infiltrating cells, comparing RIP-GP mice with RIP-GP × XCL1^–/–^ mice. Selected populations are CD103^+^XCR1^+^CD11c^+^ cells (left panel) and islet autoantigen–specific CD8 T cells (right panel) at day 7 and at day 28. (**B**) Quantification of the total number of CD11c^+^MHCII^+^ and CD103^+^XCR1^+^CD11c^+^ cells per organ as indicated. Values are displayed as mean ± SEM, and significant *P* values (Mann-Whitney *t* test) are indicated (*n* = 6–9). (**C**) Quantification of the total number of CD8 T cells and islet autoantigen–specific CD8 T cells per organ as indicated. Islet autoantigen–specific CD8 T cells have been identified by intracellular cytokine staining for IFN-γ after stimulation with the immunodominant LCMV-GP epitope GP33. Values are displayed as mean ± SEM, and significant *P* values (Mann-Whitney *t* test) are indicated (*n* = 6–9). (**D**) Organ-specific redistribution of cDC1 (left panel) and islet autoantigen–specific CD8 T cells (right panel) in the presence or absence of XCL1. Note that without XCL1, cDC1 and islet autoantigen–specific CD8 T cells are partially remained in the PDLN.

Next, we performed flow cytometry of leukocytes collected from spleen and pancreatic draining lymph nodes (PDLN) as well as of islet-infiltrating leukocytes isolated from the pancreas at day 7 or 28 after infection. We compared the total cell number per organ of RIP-GP mice and RIP-GP × XCL1^–/–^ mice and looked first at CD11c^+^MHCII^+^ cells, as general markers for cDC (Figure 4, A and B). At day 7, there was no significant difference in the number of cDC in the spleen between the 2 strains, but in the PDLN, there were more CD11c^+^MHCII^+^ cells in the RIP-GP × XCL1^–/–^ mice than in the RIP-GP mice. In contrast, in the islets, only one-fourth of cDC were found in XCL1-deficient RIP-GP mice when compared with regular RIP-GP mice (*P* = 0.011) (Figure 4B). We then gated for XCR1^+^CD11c^+^CD11b^–^ cells (cDC1) expressing CD103 (Supplemental Figure 2). Importantly, the number of such migratory cDC1 was strongly reduced in the islets of RIP-GP × XCL1^–/–^ compared with RIP-GP mice (*P* = 0.026) (Figure 4B). No significant effect of the presence or absence of XCL1 was found in the spleen, and like the findings for general cDC, more migratory cDC1 were found in the PDLN of RIP-GP × XCL1^–/–^ than regular RIP-GP mice (Figure 4B). These data indicate that, in XCL1-deficient mice, lower numbers of the potent antigen-presenting cDC1 are found in the islets during the beginning of β cell destruction. At a later time (day 28), there was still a tendency of a lower cDC1 frequency in the absence of XCL1. However, the detected difference between RIP-GP × XCL1^–/–^ and regular RIP-GP mice was not significant (Figure 4B).

### In the absence of XCL1, islet autoantigen–specific CD8 T cells are underrepresented in the islets.

Next, we analyzed the number of total and islet autoantigen–specific T cells in the islets. CD8b was used as a marker for total CD8 T cells and islet autoantigen–specific T cells were assessed by performing an intracellular cytokine staining for IFN-γ after stimulation with the immunodominant LCMV-GP epitopes GP33 and GP61 (Figure 4A and Supplemental Figure 3). At day 7 after infection, a situation similar to that found for cDC1 was observed (Figure 4C). First, in the spleen, the total CD8 T cell number was similar in RIP-GP × XCL1^–/–^ and RIP-GP mice. Second, there was a higher number of total CD8 T cells in XCL1-deficient RIP-GP mice than regular RIP-GP mice in the PDLN. Third, in the islets of RIP-GP × XCL1^–/–^ mice, the total CD8 T cell number is lower than in RIP-GP mice (*P* = 0.013). This pattern was even more pronounced for islet autoantigen–specific CD8 T cells. Importantly, there was a significant reduction of these cells in islets of XCL1-deficient RIP-GP mice compared with regular RIP-GP mice (*P* = 0.029) (Figure 4C). No significant difference in the number of islet autoantigen–specific CD4 T cells was found at both times (Supplemental Figure 4). As for total CD8 T cells, there was an accumulation in the PDLN of XCL1-deficient mice, but there was no significant difference in the spleen. At day 28 after the infection, both total and islet autoantigen–specific CD8 T cells maintained the same trend as at day 7 (Figure 4B). Thus, the absence of XCL1 causes a redistribution of cDC1 and islet autoantigen–specific CD8 T cells between PDLN and pancreatic islets (Figure 4D). Both cDC1 and islet autoantigen–specific CD8 T cells seem partially stuck in the PDLN.

### Overall fitness of islet autoantigen–specific CD8 T cells is reduced in XCL1-deficient mice.

**Figure 5:**
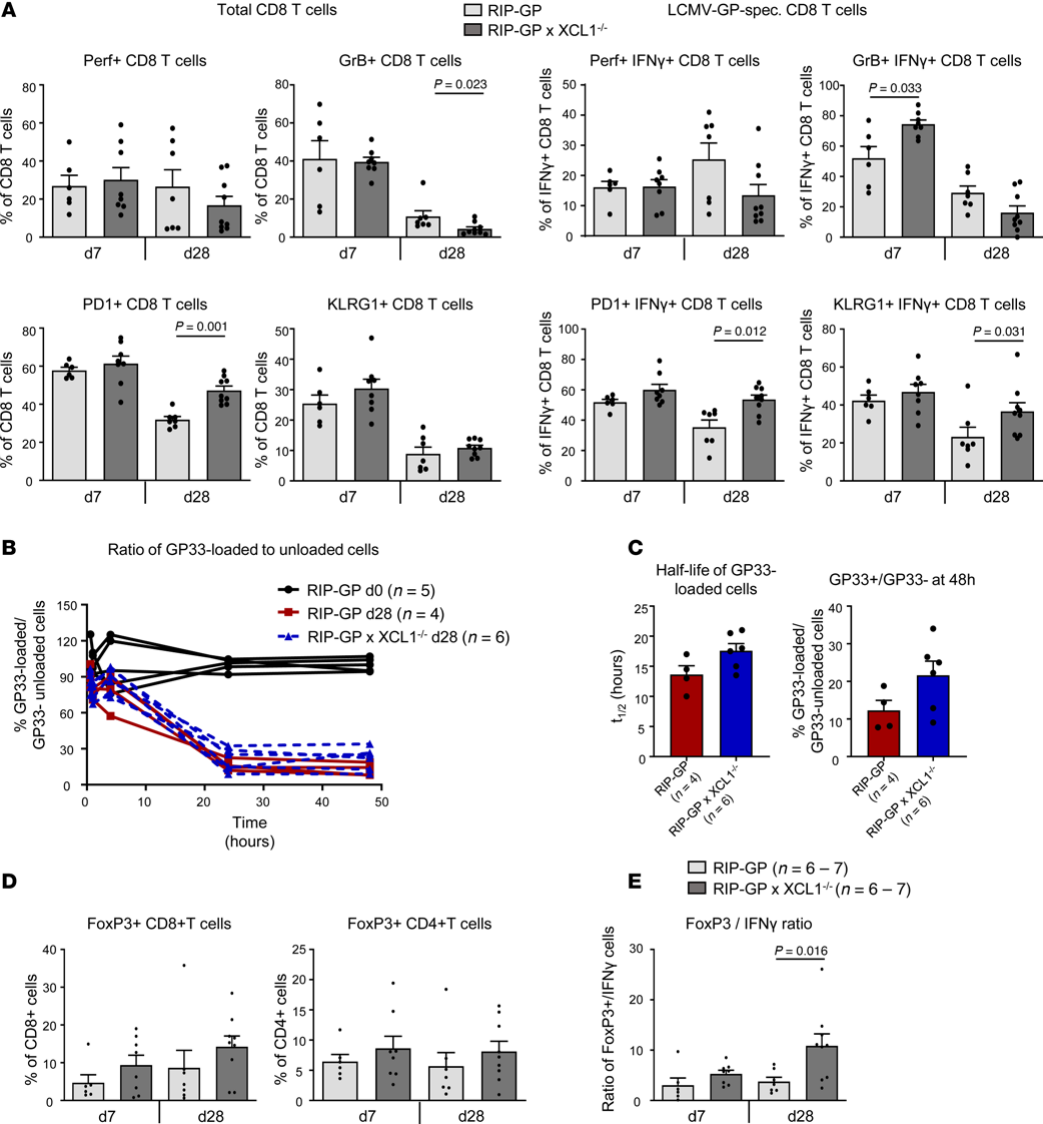
Switch to a Treg milieu in the islets of XCL1-deficient mice. (**A**) Frequencies of CD8 T cells expressing perforin (Perf), granzyme B (GrB), PD-1, or KLRG1 of total CD8 T cells or LCMV-GP33–specific CD8 T cells. Data were obtained via flow cytometric analysis of islet-infiltrating cells of RIP-GP mice and RIP-GP × XCL1^–/–^ mice at day 7 and day 28 after infection. Results are shown as mean ± SEM, and *P* values (Mann-Whitney *t* test) are indicated when significant (*n* = 7–9). (**B**) In vivo cytotoxicity assay, comparing RIP-GP uninfected mice (d0) to RIP-GP and RIP-GP × XCL1^–/–^ mice at day 28 after infection. Differently labeled GP33-loaded and unloaded target splenocytes were injected i.v. at a 1:1 ratio. At 10 minutes and 1, 4, 24, and 48 hours after injection, blood was taken, and the ratio of GP33-loaded and unloaded target cells was determined by flow cytometry. The obtained data were normalized against uninfected mice (baseline). (**C**) Calculated half-life of GP33-loaded target cell turnover (left) and different visualization of the GP33^+^/GP33^–^ ratio at 48 hours after the i.v. injection for the infected RIP-GP and RIP-GP × XCL1^–/–^ at day 28 after infection. Values are shown as mean ± SEM. Number of mice used are displayed in brackets. (**D**) Frequencies of FoxP3^+^ cells among CD8^+^ cells (left) and CD4^+^ cells (right) obtained via flow cytometric analysis of islet-infiltrating cells of RIP-GP and RIP-GP × XCL1^–/–^ mice at day 7 and day 28 after infection. Results are displayed as mean ± SEM. (**E**) Ratio of total FoxP3^+^ cells and total autoaggressive (IFN-γ^+^) CD8 T cells. Results are displayed as mean ± SEM. Number of mice and significant *P* values (Mann-Whitney *t* test) are indicated.

Clearly, the absence of XCL1 resulted in a reduced presence of cDC1 and, subsequently, also autoaggressive CD8 T cells in the islets. Thus, we assessed the overall fitness of the total and islet autoantigen–specific CD8 T cells after LCMV infection in presence or absence of XCL1, by staining for perforin and granzyme B (GrB) (cytotoxicity), programmed cell death protein 1 (PD-1), and killer cell lectin-like receptor subfamily G member 1 (KLRG1) (Figure 5A and Supplemental Figure 5A). As expected, the frequency of perforin^+^ cells was higher in islet autoantigen–specific CD8 T cells than in total CD8 T cells (Supplemental Figure 6). Furthermore, the frequency of perforin^+^ islet autoantigen–specific CD8 T cells was higher in the spleen and PDLN than in the islets at days 7 and 28 after infection (Supplemental Figure 6). In the chronic phase of T1D (day 28 after infection), the most pronounced differences between CD8 T cells in the islet microenvironment of RIP-GP × XCL1^–/–^ and RIP-GP mice were found for GrB and PD-1. XCL1-deficient mice showed fewer total GrB^+^ CD8 T cells (*P* = 0.023) and a marked increase of more functionally impaired PD-1^+^ CD8 T cells (*P* = 0.001) compared with regular RIP-GP mice (Figure 5A). Similarly, at day 28, islet autoantigen–specific CD8 T cells showed a tendency toward a lower frequency of GrB^+^ cells and displayed significantly higher frequencies of functional impaired PD-1^+^ or KLRG1^+^ CD8 T cells (*P* = 0.012 and *P* = 0.031, respectively) (Figure 5A). Apart from an increase in the frequency of GrB^+^ islet autoantigen–specific CD8 T cells, there were no significant differences in the overall fitness of CD8 T cells at day 7 after infection (Figure 5A). To further evaluate the cytotoxicity of islet autoantigen–specific T cells, we performed an in vivo cytotoxicity assay. Two sets of donor splenocytes were either loaded with GP33 or left unloaded and were differentially labeled with CFSE. These sets (GP33-loaded CFSE^lo^ and unloaded splenocytes CFSE^hi^) were mixed and injected into either RIP-GP × XCL1^–/–^ or RIP-GP mice at day 28 after LCMV infection. Uninfected RIP-GP mice were used as negative control. In vivo killing of injected target splenocytes was investigated in the blood at several times after injection. As expected in uninfected mice, no specific killing of GP33-loaded cells was observed. In contrast, in both RIP-GP × XCL1^–/–^ as well as RIP-GP mice, the GP33-loaded cells were specifically killed (Figure 5B). However, comparing the half-life of target cell elimination, a slight delay in XCL1-deficient mice was detected (Figure 5C).

### Shift to a regulatory milieu in the islets of XCL1-deficient mice.

In addition to the reduced magnitude of the autoaggressive response locally in the islets, we further evaluated its quality. RIP-GP × XCL1^–/–^ and RIP-GP mice were infected with LCMV, and the total number of FoxP3^+^ CD8 and CD4 T cells has been assessed in the islet by flow cytometry at days 7 and 28 after infection. Interestingly, in contrast to total and IFN-γ–producing islet autoantigen–specific CD8 T cells, the number of FoxP3^+^ CD4 and CD8 T cells was increased among islet infiltrating lymphocytes in XCL1-deficient RIP-GP mice both at day 7 and at day 28 (Figure 5D and Supplemental Figure 5B). Importantly, the ratio of total regulatory FoxP3^+^ T cells (CD4 and CD8) to aggressive (IFN-γ–producing) islet autoantigen–specific CD8 T cells was about 3-fold higher in the islets of XCL1-deficient mice compared with regular RIP-GP mice (*P* = 0.016) (Figure 5E). These data indicate that, in the absence of a sufficient number of cDC1, the T cell balance is tipped toward a more regulatory phenotype.

### XCL1-deficient mice are partially protected from developing T1D.

**Figure 6:**
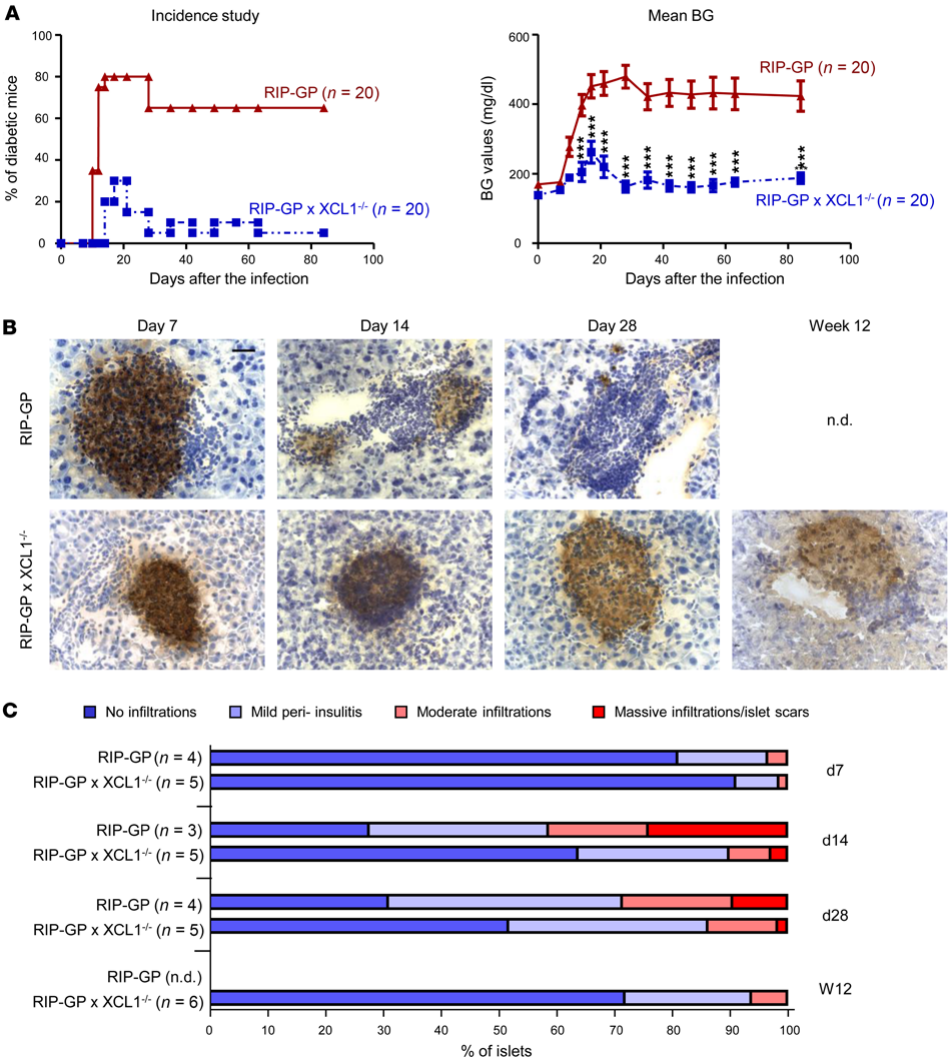
XCL1-deficient mice show less T1D incidence. (**A**) T1D incidence study comparing RIP-GP and RIP-GP × XCL1^–/–^ mice. Left panel: Percentage of diabetic mice at each time point after infection. Mice with nonfasting blood glucose (BG) levels of > 300 mg/dL were considered diabetic. Note that some mice reverted from a diabetic to a nondiabetic state over time. Right panel: Mean BG levels over time. Significant differences (2-way ANOVA) and the number of mice are indicated. (**B**) IHC staining of insulin in quick-frozen pancreas sections of RIP-GP and RIP-GP × XCL1^–/–^ mice. Representative images are shown for days 7, 14, and 28 as well as week 12 after infection. Original magnification, 40×. Scale bars: 25 μm. Note that, for RIP-GP mice, it was not possible to acquire an image at week 12 (not done; n.d.), since all the mice had to be sacrificed earlier due to severe T1D. (**C**) Mean insulitis scores determined from insulin staining shown in **B**. Islets were scored as lined out in Methods. Insulitis in RIP-GP and RIP-GP × XCL1^–/–^ mice was compared at days 7, 14, and 28 and at week 12 after infection. Number of mice are displayed in brackets.

XCL1 seems to influence the number of cDC1 in the islets, resulting in a reduced number of autoaggressive T cells but an increased number of Tregs. To evaluate whether this shift in the immune balance locally in the islets also affects T1D, we performed an incidence study. RIP-GP and RIP-GP × XCL1^–/–^ were infected with LCMV, and the blood glucose (BG) levels were measured at weekly intervals for 12 weeks. Mice with nonfasting BG levels of > 300 mg/dL were considered diabetic. Whereas RIP-GP showed a T1D incidence of 80%, the incidence was strongly reduced in XCL1-deficient RIP-GP mice (30%) (Figure 6A). Importantly, the few diabetic XCL1-deficient RIP-GP mice showed a rather mild form of T1D, since many mice reverted to a nondiabetic state 5–10 days after turning diabetic. Considering mice with remitting disease, only 1 of 20 mice displayed sustained T1D that lasted until the end of the observation time (Figure 6A). These data are also reflected in the mean BG levels that were highly elevated in regular RIP-GP mice but remained, with exception of a short elevation around days 14–21, mainly on a nondiabetic level of < 200 mg/dL in RIP-GP × XCL1^–/–^ mice (Figure 6A).

### XCL1-deficient mice display largely intact islets in pancreas tissue sections.

IHC staining for insulin of pancreas tissue isolated from RIP-GP and RIP-GP × XCL1^–/–^ at different times after LCMV infection revealed largely intact islets in XCL1-deficient mice. Representative pictures taken from pancreas tissue sections obtained at day 7, 14, and 28, as well as at week 12 after LCMV infection showed that, by day 28, the islets of RIP-GP mice were mostly destroyed, as indicated by large clusters of infiltrating immune cells and only few insulin-producing islet cells (Figure 6B). Note that most of the RIP-GP mice had to be killed before reaching the study endpoint of 12 weeks. In contrast, islets of RIP-GP × XCL1^–/–^ mice remained largely intact, with only few infiltrating cell clusters and maintained insulin production throughout the observation time. To quantify the insulitis, the islets were scored according to the degree of cellular infiltration and area of intact islet cells with insulin-production. At diabetes onset (day 14), there were more islets with moderate (insulitis score of 2) to massive (insulitis score of 3) infiltrations in the RIP-GP mice compared with RIP-GP × XCL1^–/–^ mice (Figure 6C). This trend was also visible at day 28 after infection. At week 12 after infection, when most of the RIP-GP mice had already been killed, the majority of islets of XCL1-deficient mice was still intact.

### Three-dimensional analysis of total islet content demonstrates that the absence of XCL1 prevents islet destruction.

**Figure 7:**
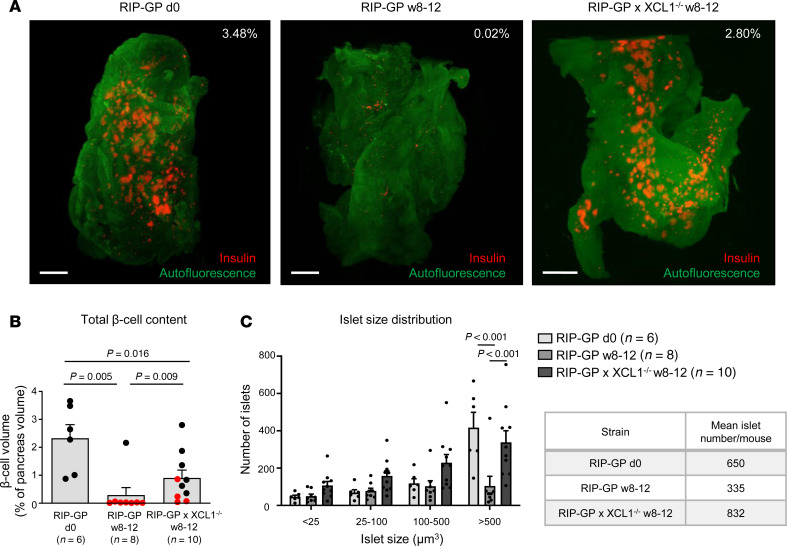
Three-dimensional analysis of total β cell content shows more functional islets in XCL1-deficient than regular RIP-GP mice. Total β cell content was determined by staining of the entire pancreas with anti-insulin antibody, followed by clearing of the tissue and scanning of the transparent pancreas by light sheet fluorescence microscopy. (**A**) Comparison between uninfected RIP-GP and LCMV-infected RIP-GP and RIP-GP × XCL1^–/–^ mice at week 8–12 after infection. Representative images are shown. Scale bars: 200 μm. The insulin-producing β cell content (red) of the representative pancreas tissues shown are indicated as a percentage of the total pancreas volume (green autofluorescence) (Supplemental Videos 1–3). (**B**) Quantification of the volume of the insulin producing cells per total pancreas volume, expressed as a percentage. Data obtained from diabetic mice are displayed in red. Values are shown as mean ± SEM. *P* values (2-way ANOVA) and numbers of mice are indicated. (**C**) Islet volume analysis: islets were separated in 4 groups according to their volume. Note that 8–12 weeks after infection, XCL1-deficient mice have larger and more insulin-producing islets left than regular RIP-GP mice. Strikingly, in comparison with uninfected RIP-GP and infected XCL1-deficient RIP-GP mice, infected regular RIP-GP mice showed a massive reduction of large (>500 μm^3^) islets. Values are shown as mean ± SEM. *P* values (2-way ANOVA) and numbers of mice are indicated.

Insulitis quantification using 2D tissue sections can be misleading. In particular, in diabetic mice with only very few remaining islets, the density of islets per section may be disproportionate; thus, the obtained data might be unreliable. Therefore, we stained the entire pancreas of uninfected RIP-GP mice as well as LCMV-infected RIP-GP and RIP-GP × XCL1^–/–^ mice for insulin at weeks 8–12 after LCMV infection (Figure 7A and Supplemental Videos 1–3). After incubation of the pancreas samples with anti-insulin antibodies, the organs were cleared and acquired with a light sheet fluorescence microscope (LSFM). The quantification of the volume of insulin producing β cells per total pancreas volume revealed a mean content of 2.33% in uninfected, healthy RIP-GP mice (Figure 7B). In LCMV-infected RIP-GP mice, the volume of insulin producing β cells dramatically decreased to only 0.29%. If only diabetic RIP-GP mice (7 of 8 in this experiment) are considered, the mean β cell volume even decreased to a minuscule 0.02%. In contrast, XCL1-deficient RIP-GP mice displayed a mean content of insulin-producing β cells of 0.91% of the total pancreas volume. Furthermore, we analyzed the size of individual islets and separated the islets in 4 groups according to their volume (large [> 500 μm^3^], medium [100–500 μm^3^], small [25–100 μm^3^], and fragments [< 25 μm^3^]) (Figure 7C). Overall, there was a marked decrease in the total number of detected islets in diabetic RIP-GP mice compared with uninfected RIP-GP mice (Figure 7C, insert). There were even more islets present in XCL1-deficient RIP-GP mice than in uninfected mice. However, considering that the overall islet volume was 2.33% versus 0.91% in uninfected RIP-GP and RIP-GP × XCL1^–/–^ mice, respectively, the higher volume in uninfected mice might largely originate from very large islets. Indeed, the highest proportion of large islets was found in uninfected mice, in which about 64% of islets had a volume of > 500 μm^3^. Importantly, besides having a low mean total number of islets, diabetic RIP-GP mice also carried only a small fraction of large islets (31%) (Figure 7C). In contrast, XCL1-deficient RIP-GP mice displayed a high total number of islets with an intermediate fraction of islets with a large volume (41%). These data indicate that, in absence of XCL1, the diabetogenic process is massively constrained resulting in a sustained content of functional islets.

## Discussion

Even after the FDA approval of the anti-CD3 antibody teplizumab, there remains an urgent need for innovative therapeutic interventions for T1D. Here, we provide evidence that the XCL1/XCR1 chemokine axis plays an important role in the immunopathogenesis of T1D and might, therefore, be an attractive novel target for immune intervention. Chemokines have been implicated in the destruction of β cells by autoaggressive T cells. In particular, CXCL10 is considered a critical inflammatory factor that drives islet infiltration. The expression of both CXCL10 and its receptor CXCR3 has been demonstrated in islets of patients with T1D as well as in diabetic mice (6, 7, 9, 19–21). Blockade of the CXCL10/CXCR3 axis successfully reduced the infiltration of islets by islet autoantigen–specific T cells and diminished the incidence of T1D in some models (6, 7). However, the efficacy of such a treatment has also been controversially discussed (22). For example, the precise timing of such an anti-CXCL10/CXCR3 therapy might be critical, since contrary to inducible animal models for T1D, the time of disease initiation is not known in patients. However, a partial depletion of T cells, as accomplished by an anti-CD3 antibody (i.e., teplizumab) treatment, induces an immune reset situation after which additional therapies may be applied, allowing a temporally better controlled regimen. Indeed, in mouse models, combination therapies with anti-CD3 and anti-CXCL10 antibodies (23) or with anti-CD3 antibody and a CXCR3 antagonist (24) have been demonstrated to exceed the efficacy of the individual monotherapies.

Many other chemokines have been demonstrated to be expressed in patients with T1D and in experimental animals (20, 21). However, the precise kinetic of their expression has not yet been assessed in detail. We therefore used the inducible RIP-GP model and dissected the islet microenvironment by laser capture microscopy at several times before and after T1D initiation and analyzed the gene expression by gene array (4). For the present study, we focused on the XCL1/XCR1 chemokine axis since both ligand and receptor were strongly upregulated during the course of islet destruction and remained high in both RIP-GP mice and NOD mice. In addition, XCR1 is expressed exclusively on the cDC1 subtype of DC, which is characterized by high T cell activation properties and their strong capacity for cross-presentation (11, 12, 14). XCR1-expressing cells were also found to be increased in the islet microenvironment of individuals with autoantibodies and with a T1D diagnosis. Interestingly, the frequency of XCR1-expressing cells was also increased in the islets of nondiabetic individuals with known T1D familiarity. Thus, these individuals might have an increased susceptibility or might be on the verge of soon developing autoantibodies. Even if the latter assumption is difficult to prove, the findings in human pancreas samples underline the importance of the XCL1/XCR1 axis and further suggest an involvement in T1D pathogenesis in humans. In contrast, almost no cells producing XCL1 mRNA were detected. One reason might be the low abundance of CD8^+^ T cells found in the sections. It has been previously demonstrated that the number of total CD8 T cells, and of islet autoantigen–specific CD8 T cells in particular, is lower in human islets than in islets of RIP-GP or NOD mice (25). Other possible explanations include the transient nature of chemokine expression and RNA degradation over time.

Since there are no reliable inhibitors for the XCL1/XCR1 axis available at the moment, we introduced XCL1-deficient mice to the RIP-GP model. Such RIP-GP × XCL1^–/–^ mice displayed a dramatically decreased T1D incidence compared with regular RIP-GP mice. In parallel, XCL1 deficiency resulted in a strongly reduced insulitis. To better quantify the remaining content of functional β cells, the entire pancreas was stained for insulin and scanned with a LSFM. This technique allows, on the one hand, a 3D visualization of the islet content (Figure 7 and Supplemental Videos 1–3) and, on the other hand, a determination of the total volume of functional, insulin-producing β cells. In diabetic RIP-GP mice, the total functional β cell content was reduced to 12% of the content found in uninfected mice. In contrast, in the absence of XCL1, the content was only reduced to 39%, which was sufficient to maintaining a healthy BG level. During T1D, it seems that predominantly large islets are disrupted and reduced in volume, whereas in uninfected mice, the total functional β cell content mainly originates from large islets. The islet size distribution observed in uninfected mice is largely maintained in LCMV-infected RIP-GP × XCL1^–/–^ mice, indicating that XCL1 might pave the path for a subsequent islet disruption by cDC1 and other leukocytes, including autoaggressive T cells.

XCL1 and/or XCR1 have been found in context of T1D mainly in screenings for inflammatory factors with other chemokines (6, 20, 21, 26, 27). However, there were no follow-up studies regarding their significance in the immunopathogenesis of T1D. Nevertheless, XCL1 has been demonstrated to play an important role in intestinal immune homeostasis (28) and in T cell priming in the skin (29). Indeed, stimulation of skin XCR1^+^ cDC1 has been found to be beneficial for the treatment of melanoma in mice (29).

Whereas the presence of resident and infiltrating macrophages has been clearly demonstrated in human islets by mass spectrometry (30), the situation is not that clear for the individual DC subtypes (31). In contrast, the role of cDC1 that uniquely express XCR1 and are attracted by XCL1 have been investigated in context with T1D in mouse models. CD103^+^ cDC1 are found at an increasing number from week 3 to 4 of age in NOD mice (32), and they play a crucial role in the pathogenesis of T1D, since Batf3-deficient NOD mice lacking CD103^+^ cDC1 in the islets and PDLN are protected from T1D (33). However, CD103, which is an integrin (αEβ7) that binds to E-cadherin, seems to have only a minor effect on the development of T1D, since CD103-deficient RIP-GP mice generated a similar anti–islet antigen T cell response and T1D incidence as normal RIP-GP mice (34). Thus, due to the abundance of various other cell adhesion proteins and chemokine axes, the integrin CD103 itself seems to be redundant in the inflammatory milieu of the islet microenvironment. Furthermore, CD103 is also found on T cells. In fact, 50%–70% of CD8 T cells expressed CD103 in the islets of patients with T1D (35). Therefore, CD103 deficiency might have differential effects on cDC1 and T cells.

The XCL1/XCR1 axis has been shown to be important for the cluster formation between DC and T cells and subsequently for the effective activation of aggressive T cells (14). Due to the fact that, upon activation, T cells express XCL1 and cDC1 express CXCL10, a mutual attraction of these cell types might lead to cluster formation and suggests that both XCL1 and CXCL10 are of particular importance in the potentiation of T1D. Recently, it has been demonstrated that Tregs can act on aggressive CD8 T cells via suppression of cDC1-mediated T cell priming (36). Thus, functional cDC1 seem to be required in sufficient numbers for an effective activation of aggressive T cells. In our study, mechanistically, XCL1 was required to attract sufficient numbers of cDC1 to the islet microenvironment, which subsequently recruit and activate islet autoantigen–specific T cells. Importantly, fewer islet autoantigen–specific CD8 T cells were found in the islets of XCL1-deficient mice, and those cells displayed a reduced overall fitness compared with autoaggressive CD8 T cells in regular RIP-GP mice. In contrast, the frequency of FoxP3^+^ CD4 and CD8 T cells was increased in XCL1-deficient mice. Thus, in absence of XCL1, the T cell balance was tipped toward a more regulatory milieu.

Therapeutically, our findings indicate that a blockade of the XCL1/XCR1 axis might be beneficial for patients with T1D. In contrast to cell depletion therapy, an XCL1/XCR1 interference can be regarded as a rather mild immunosuppressive intrusion. Currently a repetitive administration of teplizumab is not considered due to safety concerns. Thus, combination therapies are being considered to prolong the beneficial effect (24). Blocking the XCL1/XCR1 axis in parallel could be such a combination therapy. Thereby, the lack of sufficient numbers of cDC1 in the islet microenvironment might reduce the activation and regeneration of autoaggressive CD8 T cells and the de novo infiltration of the islets. In addition, the presence of a higher frequency of Tregs might further prevent this activation. Furthermore, a XCL1/XCR1 axis blockade might be an interesting addition to the administration of tolerogenic DC (37). A reduced cDC1 frequency paired with an increased presence of islet autoantigen–pulsed tolerogenic DC might further tip the immune balance toward a regulatory milieu that would strongly favor the generation of islet autoantigen–specific regulatory T cells. We have tested 2 different anti-XCL1 antibodies that have been previously used for in vivo blockade of the XCL1/XCR1 axis. One antibody distributed by R&D systems has been successfully used to reduce the XCL1-mediated cDC1 recruitment into the tumor microenvironment in mice (14). The other antibody we have tested has been successfully used to reduce the severity of experimental autoimmune encephalitis (EAE) and collagen-induced arthritis (CIA) in mouse models (38). However, this antibody predominantly blocks the interaction of XCL1 and α9-integrin rather than XCR1 (38). Unfortunately, there was no significant change in T1D incidence and severity in RIP-GP mice that have been treated with either of the 2 antibodies compared with mice receiving isotype-matched control antibodies (data not shown). However, since these antibodies showed also no inhibitory effect on the migration of in vitro–differentiated cDC1 in migration assays, we cannot exclude that a therapeutic interference with the XCL1/XCR1 axis might have an effect on the pathogenesis of T1D. Thus, the generation of a reliable inhibitor is required to further prove the importance of the XCL1/XCR1 axis.

It has been demonstrated that CD8 T cells recruit cDC1 directly via XCL1 secretion (39). CD8 T cell–secreted XCL1 might therefore be important in maintaining clusters of infiltrating cells that activate each other perpetually. In this context, targeting both the CXCL10/CXCR3 as well as the XCL1/XCR1 axes might be another promising approach for a combination therapy. Parallel interference with the migration of cDC1 and T cells to the islet microenvironment might thereby disrupt pathogenic immune cell clusters, strongly diminish T cell activation, and ablate β cell destruction.

## Methods

### Sex as a biological variable.

Our study examined male and female animals, and similar findings are reported for both sexes.

### Mice and virus.

RIP-GP transgenic mice were generated and screened by PCR as previously described (5, 40). XCL1^–/–^ mice were generated as previously described by Dorner et al. (11) and have been backcrossed to C57BL/6 mice for more than 10 years. Here, the XCL1^–/–^ mice were crossed with RIP-GP mice to study T1D (RIP-GP × XCL1^–/–^). LCMV Armstrong clone 53b was produced as described previously (40). Mice were infected with a concentration of 1 × 10^4^ plaque-forming units of LCMV. Nonobese diabetic (NOD) mice were purchased from Charles River Laboratories. BG was measured in weekly intervals using a CodeFree glucometer from SD Biosensor Inc. Animals with nonfasting BG concentrations higher than 300 mg/dL were considered diabetic.

### Human samples.

Human pancreas paraffin slides were obtained from the HPAP (41), a component of the Human Islet Research Network. These samples were obtained by donors with different disease stages; in particular, 8 of them were healthy donors, 5 of them were classified as islet autoantibody positive (Aab^+^) since 1 or more islet autoantibodies could be found in their blood but HbA1c and since C-Peptide (C-Pep.) values were normal, and 6 of them were affected by T1D. The 8 ND were further split in 2 groups: 5 nondiabetic (ND) and 3 nondiabetic but with known T1D familiarity (T1D familiarity). Detailed information about organ donors is reported in Supplemental Table 1. The human pancreas sections have been tested for RNA integrity sufficient for the RNAScope duplex detection method (ACD/Bio-Techne) using positive control probe sets provided by the supplier. For the quantification of XCR1-expressing cells in and around the islets, all the islets detected any given pancreas section have been counted. Depending on the size and the location of the small piece of pancreas tissue, the number of detectable islets varied from 6 to 63 islets per section.

### Islet laser dissection and gene array.

Mouse pancreata were obtained on days 0 (uninfected), 1, 3, 7, 10, 14, and 28 after infection. Islet microenvironments were collected from 3 μm frozen pancreatic sections by laser capture dissection (LCM) using an Axiovert 200M laser-dissection microscope (Zeiss) as previously described (4). After RNA extraction using a RNeasy Micro Kit (Qiagen) and quality control using RNA 6000 Nano Chips (Agilent Technologies), the total RNA amplification and cDNA labeling was done using standardized protocols (Ovation Pico WTA System V2 amplification kit and Encore Biotin Module labelling kit; NuGEN). Microarray hybridization to GeneChip Mouse Gene 1.0 ST V1 arrays (Affymetrix) was performed according to the Affymetrix protocol. The data have been deposited in the NCBI Gene Expression Omnibus and are accessible through GEO series accession no. GSE229287 (https://www.ncbi.nlm.nih.gov/geo/query/acc.cgi?acc=GSE229287).

### Real-time PCR/gene expression analysis.

Expression of selected genes was evaluated by qPCR (4). Briefly, total RNA (input: 500 pg to 50 ng) was amplified using Ovation Pico SL WTA System V2 amplification kit (NuGEN) following manufacturer’s instructions. Reverse transcription was performed with MMuLV reverse transcriptase (Promega). Real-time PCR was performed using TaqMan gene expression assays (Applied Biosystem) with 100 ng cDNA for each reaction in duplicate. The following TaqMan probes were used: XCL1 (Mm00434772_m1) and XCR1 (Mm00442206_s1). Data were normalized using GAPDH housekeeping gene (Applied Biosystems; catalog 4352339E), and subsequent analysis was performed using 2^–ΔΔCT^ method.

### RNAScope.

Pancreata together with the PDLN were dissected, immerged in a solution of buffered paraformaldehyde 4% and incubated overnight on a shaker at room temperature. The following day, samples were dehydrated using Leica TP 1020 Tissue Processor and were then embedded in paraffin. Slides (4 μm) were cut, and ACDBio RNAScope 2.5 HD Duplex Manual Assay kit (ACD/Bio-Techne) was used on the sections, following the manufacturers protocol. The incubation time of “target retrieval” and “protease plus” for mouse pancreas slides was reduced to 11 minutes and 15 minutes, respectively. The obtained human pancreas slides were incubated for 15 minutes for “target retrieval” and for 30 minutes in the “protease plus” step. The different probes were detected in channel 1 (C1) through an enzymatic reaction via horseradish peroxidase (HRP) to develop a blue color and in C2 through an alkaline phosphatase reaction, developing a red color. The probes were XCL1 (catalog 507791-C2, red) and XCR1 (catalog 562371, blue) for mouse samples and XLC1 (catalog 562471-C2, red) and XCR1 (catalog 542041, blue) for human samples. Nuclei were counterstained with Mayer’s hematoxylin. Images were acquired using an Axioscope 2 microscope (Zeiss) with a 40× or 63× oil objective.

### IHC staining.

Dissected pancreata with PDLN were immerged in Tissue-Tek OCT and quick frozen on dry ice. Tissue sections (7 μm) were cut and then fixed in ethanol or ethanol/acetone (1:1) at –20°C. The sections were blocked with 10% FCS in PBS. Primary antibodies used were rat anti-CD8b (1:100, BioLegend, 126602), goat anti-CD103 (1:300, R&D Systems, AF1990), Armenian hamster anti-CD11c (1:200, Invitrogen, 14-0114-82), and rabbit anti-insulin (1:4,000, Abcam, ab181547). As secondary antibodies, biotinylated anti-rat (1:500, Vector, VEC-BA-4001), biotinylated anti-goat (1:400, Vector, VEC-BA-5000), biotinylated anti–Armenian hamster (1:400, eBioscience, 13-4113-85), and biotinylated anti-rabbit (1:500, Vector, VEC-BA-1000) were used. After ABC complex (Vector Laboratories), DAB substrate was used to develop color, and nuclei were counterstained with hematoxylin. Images of pancreas sections were acquired with an Axioscope 2 microscope (Zeiss) with a 40× objective. The software BZII Analyzer (Keyence) was used for the quantification of the IHC images.

### Immunofluorescence staining.

Samples were processed as for IHC. Primary antibodies used were goat anti–mouse CD103 (1:300, R&D Systems, AF1990) and Armenian hamster anti-CD11c (1:200, Invitrogen, 14-0114-82). As secondary antibodies, Cy3-conjugated anti-goat (1:50, Jackson ImmuneResearch, 127-095-099) and biotinylated anti–Armenian hamster (1:400, eBioscience, 13-4113-85) were used, followed by an incubation with FITC-conjugated streptavidin (1:500, BioLegend, 405201). In addition, DAPI (1:5,000, MilliporeSigma) was added to the mix to stain for the nuclei. Images of pancreas sections were acquired with a confocal microscope LSM510 (Zeiss).

### Isolation of islet infiltrating leukocytes.

Islets were isolated as previously described (42). Briefly, pancreata were dissected, removing PDLN, and were injected with 1.2 U/mL collagenase P in RPMI 1640 + GlutaMax. Then, 7 mL of warm RPMI were added. Samples were digested for 30 minutes at 37°C. Digestion was then stopped by adding cold RPMI, and pancreata were disaggregated, shaken in the tube for 1 minute, and then squeezed through a kitchen sieve. After spinning down the samples, a gradient was applied containing Ficoll-Paque PLUS overlayed with warm RPMI. Islets were isolated from the interface between the 2 phases and combined with the hand-picked islets from the pellet. To avoid the exocrine part of the pancreas and to isolate only the islet-infiltrating leukocytes, CD45 Microbeads (Miltenyi Biotec) were applied following the manufacturers protocol to enrich CD45^+^ cells.

### Isolation of leukocytes from spleen and PDLN.

Spleens and PDLN were collected and squeezed through 70 μm cell strainers. After centrifugation (550*g* for 5 minutes), the pellets were resuspended in RPMI and stored on ice until further use. For splenocytes, an erythrocytes lysis step was performed, adding 2 mL of 0,83% NH_4_Cl/H_2_O for 2 minutes. The lysis was stopped with RPMI.

### Flow cytometry.

Single-cell suspensions of leukocytes isolated from spleen, PDLN, or pancreas were stimulated overnight with the immunodominant LCMV peptides GP33 (10 μg/mL, GenScript) for CD8 T cells and GP61 (10 μg/mL, GenScript) for CD4 cells in the presence of Brefeldin A (1 μg/mL, MilliporeSigma). Cells were stained for surface antigens with the following antibodies: BV510-conjugated anti-CD8b (clone YTS156.7.7, BioLegend, 126631), PE/Cy7-conjugated anti-CD11c (clone N418, BioLegend, 117318), eFluor450-conjugated anti-MHCII (clone M5/114.15.2, Invitrogen, 48-5321-82), APC/Cy7-conjugated anti-CD11b (clone M1/70, BioLegend, 101226), APC-conjugated anti-CD103 (clone M290, BD Biosciences, 562772), and BV510-conjugated anti-XCR1 (clone ZET, BioLegend, 148218). They were then fixed, permeabilized with PFA/saponin solution, and stained for intracellular targets using the following antibodies: V450-conjugated anti–IFN-γ (clone XMG1.2, BD Biosciences, 560661), PE-conjugated anti-FoxP3 (clone FJK-16S, Invitrogen, 12-5773-82), PE-conjugated anti-GrB (clone NGZB, Invitrogen, 128898-80), and APC-conjugated anti-perforin (clone S16009A, BioLegend, 154403). Samples were acquired with a MACSQuant flow cytometer (Miltenyi Biotec) and analyzed with FlowLogic 7.3 (Inivai Technologies).

### Insulitis scoring.

Insulitis was scored according to the following system: 0: Very minor or no insulitis, only very few infiltrating cells; 1: mild to moderate insulitis, 25%–50% infiltrations, large parts with intact β cells; 2: considerable insulitis, 50%–75% infiltrates, still some parts with intact β cells; 3: massive insulitis, 75%–100% infiltrates, only few remaining β cells producing insulin, islet scar.

### Whole-pancreas staining.

This protocol is a modified version of the iDISCO+ protocol (iDisco Protocol; https://idisco.info/idisco-protocol/). Mice were sacrificed with an overdose of isoflurane and immediately perfused through the heart with 10 mL buffered paraformaldehyde (4%). The pancreas was removed and incubated on a shaker for 2 hours at room temperature in buffered paraformaldehyde (4%). The sample was then washed with PBS/Triton X-100 (0.2%) 2 times for 1 hour and incubated on a wheel overnight with the pretreatment solution 1 (PBS containing 0.2% Triton X-100 and 20% DMSO) at 37°C. The solution was changed to pretreatment solution 2 (PBS containing 0.1%, Triton X-100, 0.1% deoxycholate, 0.1% NP40, and 20% DMSO) for another overnight incubation on a wheel. The pancreas was washed 2 times for 1 hour with PBS/Triton 0.2% and permeabilized with a permeabilization solution (PBS containing 0.2% Tween 20, 5% DMSO, 2.3% glycine, and 0.02% NaN_3_) for 2 days at 37°C shaking. Blocking was performed with a filtered blocking solution (PBS containing 0.2% Triton X-100, 10% DMSO, and 6% donkey serum) for 2 days at 37°C shaking. Then the sample was incubated with the first antibody (rabbit anti-insulin; Abcam, ab181547). The antibody was diluted in first antibody buffer (PBS containing 5% DMSO, 3% donkey serum, 0.2% Tween 20, and 10 μg/mL heparin), and the antibody concentration was increased every day (1:3,200, 1:2,000, 1:1,600, 1:800). The sample was incubated while shacking at 37°C during the day and centrifuged at 600*g* overnight for better tissue penetration. The pancreas was washed 4 times for 1 hour and 1 time overnight with PBS Tween with Heparin (PTwH) buffer (PBS containing 0.2% Tween-20 and 10 μg/mL heparin). The secondary antibody incubation was performed in a similar way as the first one. The antibody used was a Cy3-conjugated anti-rabbit antibody (Jackson Immune Research, 711-167-003) at increased concentrations of 1:800, 1:600, 1:400, 1:200 in secondary antibody buffer (PBS containing 3% donkey serum, 0.2% Tween 20, and 10 μg/mL heparin). After several washing steps with PTwH as described above, the pancreas was embedded in 1.3% low-melting agarose (in H_2_O). After gel solidification, the sample was dehydrated, by incubating with tetrahydrofuran (MilliporeSigma) at increasing concentrations (in H_2_O) while shaking (50% overnight at 4°C, 70%, 80%, and 100% for 2 hours, respectively, at room temperature and then 100% overnight at 4°C). The lipids were removed from the sample with dichloromethane (MilliporeSigma) with an incubation of 30 minutes at room temperature while shaking. The sample was then cleared with dibenzyl ether (MilliporeSigma) overnight while shaking and then stored in dibenzyl ether (MilliporeSigma) until acquisition.

### Whole pancreas acquisition and quantification.

The whole stained pancreas was acquired in dibenzyl ether with the ultramicroscope Olympus MVX10, from LaVisionBiotec, with SuperKExtreme from NKTPhotonics as lasers and Neo 5.5 CMOS. The program used for the acquisition was Imspector Pro. Scanned images were analyzed with the Imaris 9.8.2. software.

### In vivo cytotoxic assay.

In vivo cytotoxic assay was performed as previously described (43). Briefly, splenocytes isolated from donor mice were used as target cells. One half of the splenocytes was coated with 2 μg/mL of LCMV-GP33 peptide overnight, and the other half was incubated with culture medium only. The LCMV-GP33–coated splenocytes were labeled with CFSE (Invitrogen) at a concentration of 0.5 μM CFSE (CFSE^lo^) and uncoated splenocytes were labeled with 5 μM (CFSE^hi^). The 2 subgroups were then mixed 1:1 at a concentration of 5 × 10^6^ cells/mL each. The mixture was then injected i.v. into the recipient mice. As recipient mice, either uninfected RIP-GP, infected RIP-GP, or infected RIP-GP × XCL1^–/–^ mice at day 28 after the infection were used. Blood was taken at several times after splenocyte injection (10 minutes and 1, 4, 24, and 48 hours), and the ratio between the 2 CFSE-labeled target cell populations was calculated. The calculated ratio was then normalized to the ratio obtained for the 10-minute point.

### Plaque assay.

As previously described (44), MC57G cells (C57BL/6 fibroblast cell line) were incubated with serially diluted homogenized spleens of infected mice sacrificed at day 3 and day 7 after infection, overlaid with a monolayer of methylcellulose. Cells were then fixed with 4% buffered paraformaldehyde, and the formed plaques were stained first with a rat anti-LCMV antibody (VL-4, 2 mg/mL) and, second, with peroxidase–conjugated AffiniPure goat anti-rat IgG (H+L) (Jackson ImmunoResearch Laboratories). DAB was used as chromogen; the stained plaques were counted, and the titer was calculated according to the following formula:


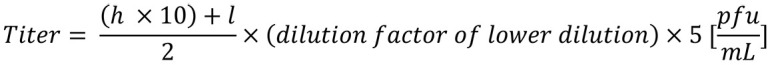


where “h” indicates number of foci in the well with the higher dilution (fewer foci), “l” indicates the number of foci in the well with the lower dilution (more foci), and “× 5” indicates that 200 μL were added; therefore divided by 200 × 1,000.

### Statistics.

The statistical analysis of the experiments was done using Mann-Whitney 2-tailed *t* test and 2-way ANOVA with Bonferroni as a post hoc test (GraphPad Prism software version 5.02). A *P* value of less than 0.05 was considered significant.

### Study approval.

Animal experiments were approved by the local Ethics Animal Review Board (V54–19c20/15-FU/1265 and FU/2049).

### Data availability.

Regarding Figure 1, the raw gene array data have been deposited in the NCBI Gene Expression Omnibus and are accessible through GEO series accession no. GSE229287. Regarding Figures 2–7 and Supplemental Figure 1, the raw data for all data points shown in graphs, and values behind any reported means are available in the Supporting Data Values file.

## Author contributions

CT designed the experiments, generated raw data, analyzed and interpreted data, and helped draft the manuscript. CB, DP, EB, M Bayer, GKB, M Bachmann, and EH, carried out the experiments and analyzed the data. JP, RPB, GJG, MRB, and RAK provided material or mice and helped design the experiments and draft the manuscript. UC designed the study, generated raw data, analyzed and interpreted data, and drafted the manuscript. All authors helped to critically revise the intellectual content of the manuscript and approved the final submission. UC is the guarantor of this work and takes responsibility for the contents of the article.

## Supplementary Material

Supplemental data

Supplemental video 1

Supplemental video 2

Supplemental video 3

Supporting data values

## Version 1 02/27/2025

In-Press Preview

## Version 2 04/08/2025

Electronic publication
