# Supplementary material for: The XCL1/XCR1 axis is upregulated in type 1 diabetes and aggravates its pathogenesis
Source: JCI Insight. 2025 Feb 27;10(7):e178743. doi: 10.1172/jci.insight.178743 (PMC11981631; doi:10.1172/jci.insight.178743)
Supplement: Supplemental data [file jciinsight-10-178743-s201.pdf]

Supplemental Table 1. Detailed information about organ donors, obtained through the HPAP programme.

Donor	Sample collection	Pancr. region	State	Fam.	Age	Sex	Disease Duration (years)	HbA_{1c} (%)	C-Pep. (mmol / l)	Aabs
HPAP-034	12.2018	Tail	ND		13	M		5.2	12.7	-
HPAP-072	10.2020	Tail	AAb+		19	M		5.6	4.37	GAD
HPAP-087	02.2021	Tail	T1D		15	F	8	10.4	0.02	IAA
HPAP-095	06.2021	Tail	ND	Yes	23	F		4.9	4.13	-
HPAP-099	06.2021	Tail	ND		28	F		5	6.62	-
HPAP-102	07.2021	Tail	T1D		18	M	6	6.7	0.07	IAA
HPAP-107	10.2021	Tail	AAb+		15	M		5.3	5.49	GAD, IA-2, ZnT8
HPAP-110	12.2021	Tail	ND		31	M			7.75	-
HPAP-114	01.2022	Tail	AAb+		21	F		5.3	11.45	GAD
HPAP-123	04.2022	Tail	T1D		25	M	3	9.7	0.07	GAD, IAA, ZnT8
HPAP-135	11.2022	Body	T1D		18	M	3	14.7	0.25	-
HPAP-092	04.2021	Tail	AAb+		21	M		5.6	15.35	GAD
HPAP-136	11.2022	Body	ND		29	M		5.4	7.14	-
HPAP-137	12.2022	Tail	T1D		23	M	2.5	16.1	0.12	-
HPAP-139	12.2022	Tail	ND		22	M		5.2	9.43	-
HPAP-140	01.2023	Tail	ND	Yes	29	F		4.7	10.4	-
HPAP-146	03.2023	Tail	ND	Yes	27	M		5.5	9.56	-
HPAP-148	04.2023	Tail	AAb+		7	M		5.3	4.62	GAD, IA-2
HPAP-151	05.2023	Tail	T1D		31	M	3.5	14	0.02	IAA

ND: Non-diabetic individuals; AAb+: Individuals with autoantibodies against at least one islet autoantigen; T1D: Individuals with type 1 diabetes; Fam.: familiarity; Pancr.: Pancreas.

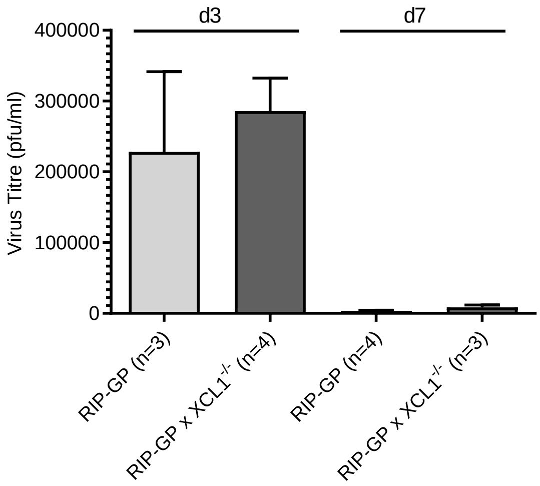

Supplemental Figure S1: XCL1-deficient mice do not have an altered immune system. - Titre (pfu/ml) of LCMV found in the spleen of RIP-GP and RIP-GP x XCL1$^{-/-}$ mice at day 3 and day 7 after infection as determined by a LCMV plaque assay. Number of mice is indicated in brackets. Note that there is no significant difference between XCL1-deficient and regular RIP-GP mice and that at day 7 almost no virus is left.

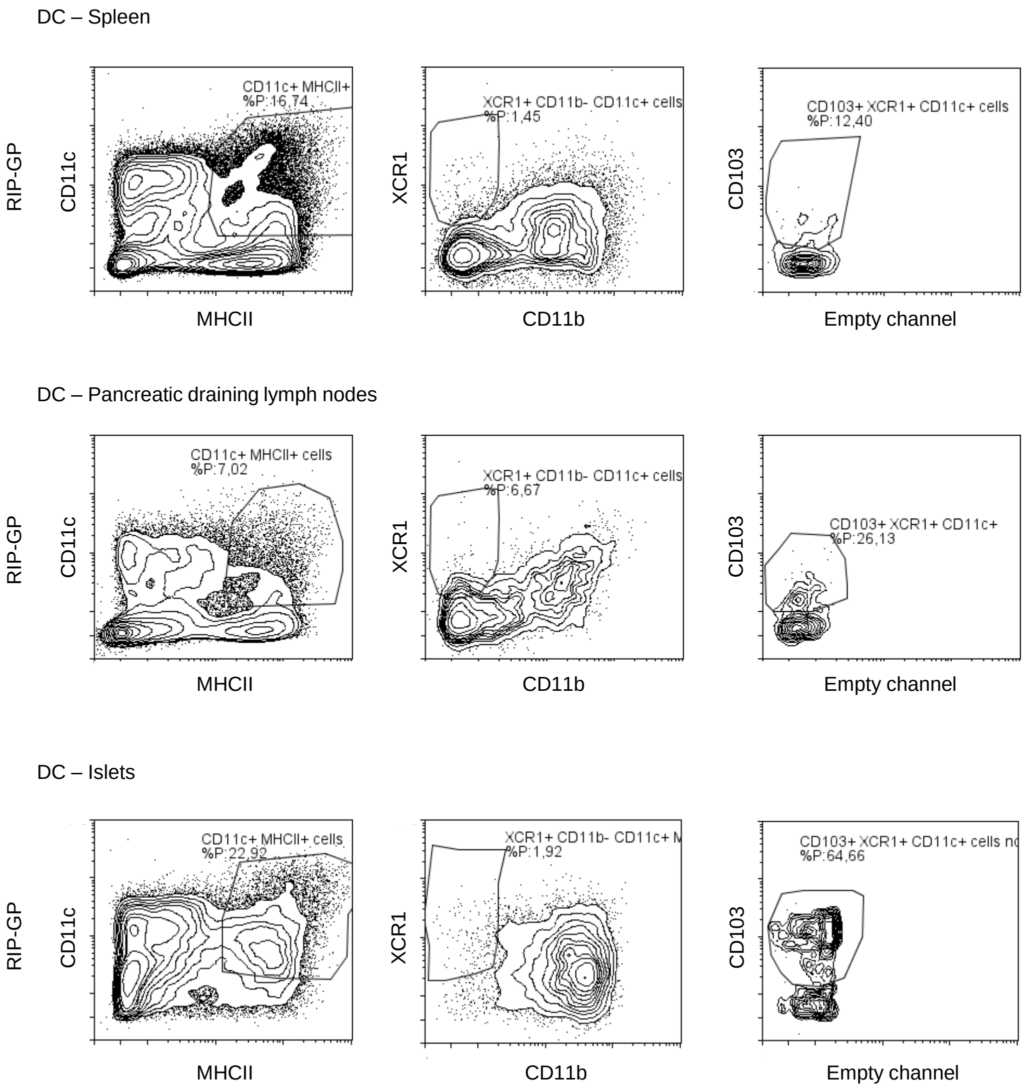

Supplemental Figure S2: Gating strategy for DC in spleen, pancreatic draining lymph nodes and islet infiltrating cells (islets). - Representative DC analysis of dot plots obtained via flow cytometry. The analysis is shown for a RIP-GP mouse at day 7 after LCMV-infection.

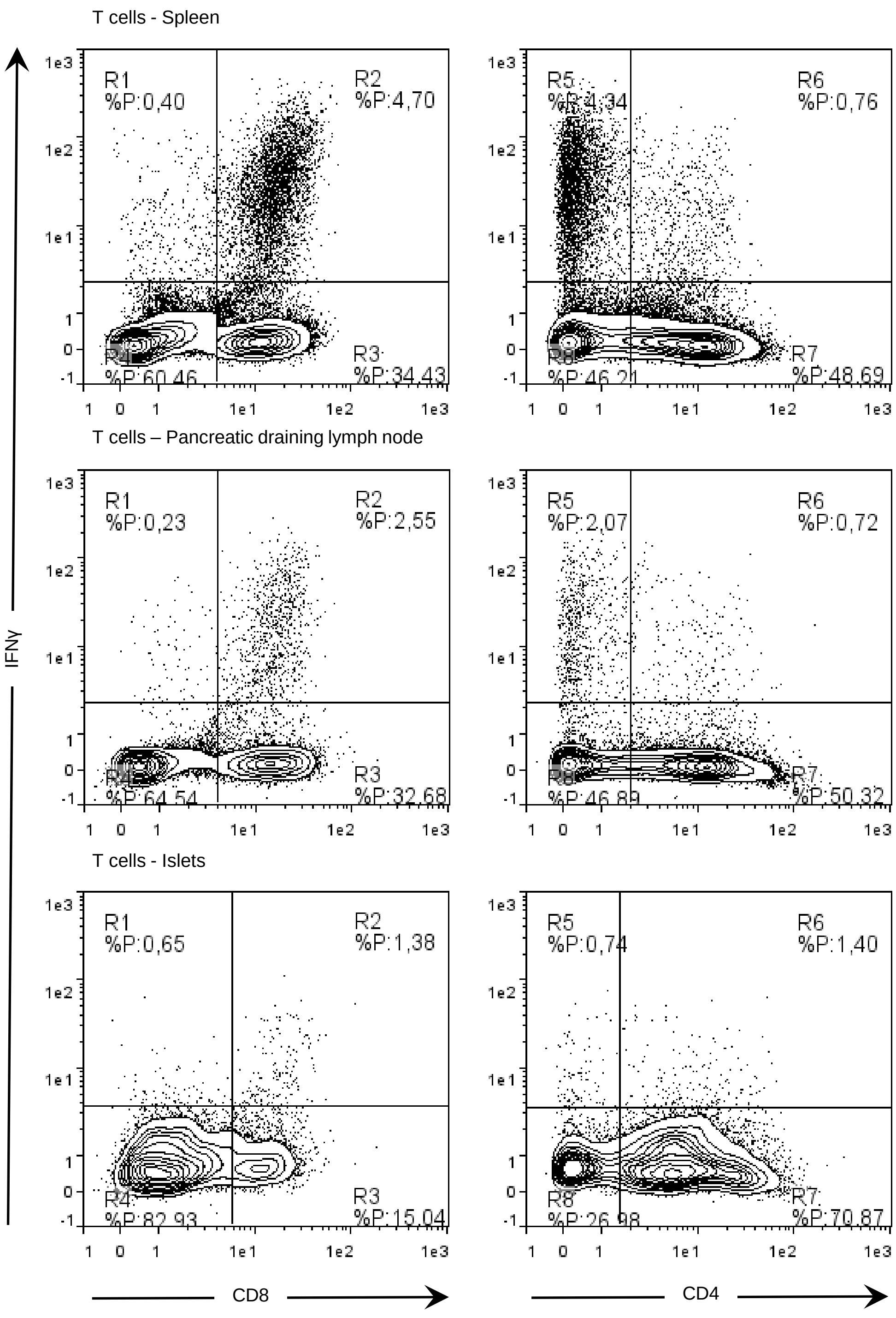

Supplemental Figure S3: Gating strategy for T cells in spleen, pancreatic draining lymph nodes and islets infiltrating cells (islets). - Representative T cell analysis of dot plots obtained via flow cytometry. The analysis is shown for a RIP-GP mouse at day 7 after LCMV infection. Islet autoantigen-specific T cells are in the upper right quadrants.

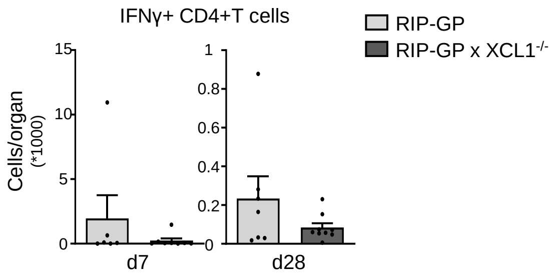

Supplemental Figure S4. Antigen-specific CD4 T cells seem to decrease in RIP-GP x XCL1$^{-/-}$ islets. Absolute number of LCMV-GP33 specific (IFNγ-producing) CD4+ cells among the islet infiltrating cells obtained via flow cytometric analysis at day 7 and day 28 after infection, comparing RIP-GP mice with RIP-GP x XCL1$^{-/-}$ mice. Values are displayed as mean ± SEM and significant p-values are indicated (n=6-9).

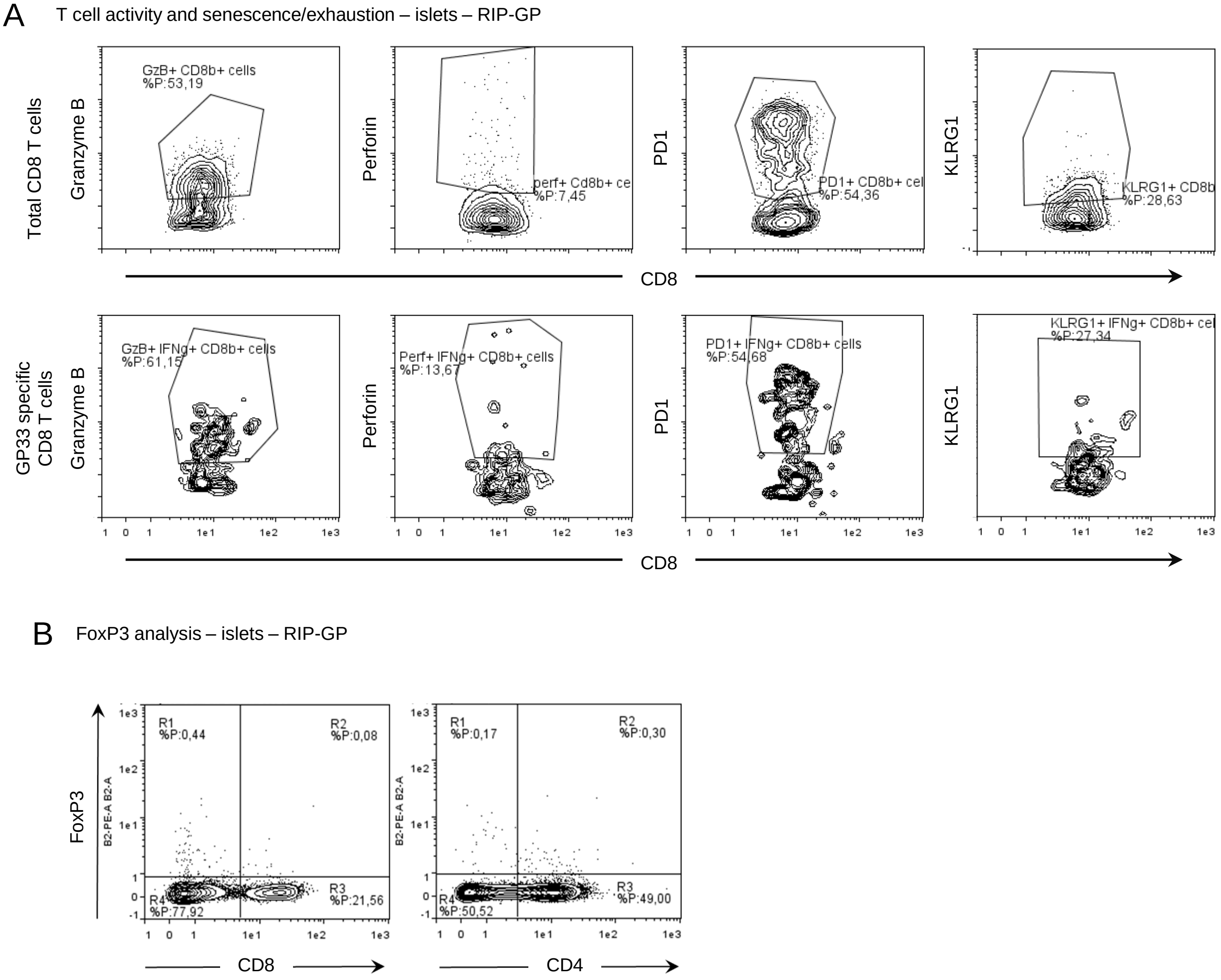

Supplemental Figure S5. Gating strategy of T cell activity for islets infiltrating cells (pancreas). *A*. Dot plots representing granzyme B, perforin, PD1 and KLRG1 analysis both of total CD8 T cells and GP33 specific T cells isolated from the islets of a RIP-GP mouse at day 7 after the infection. *B.* FoxP3 cells dot plot analysis of a RIP-GP mouse at day 7 after the infection.

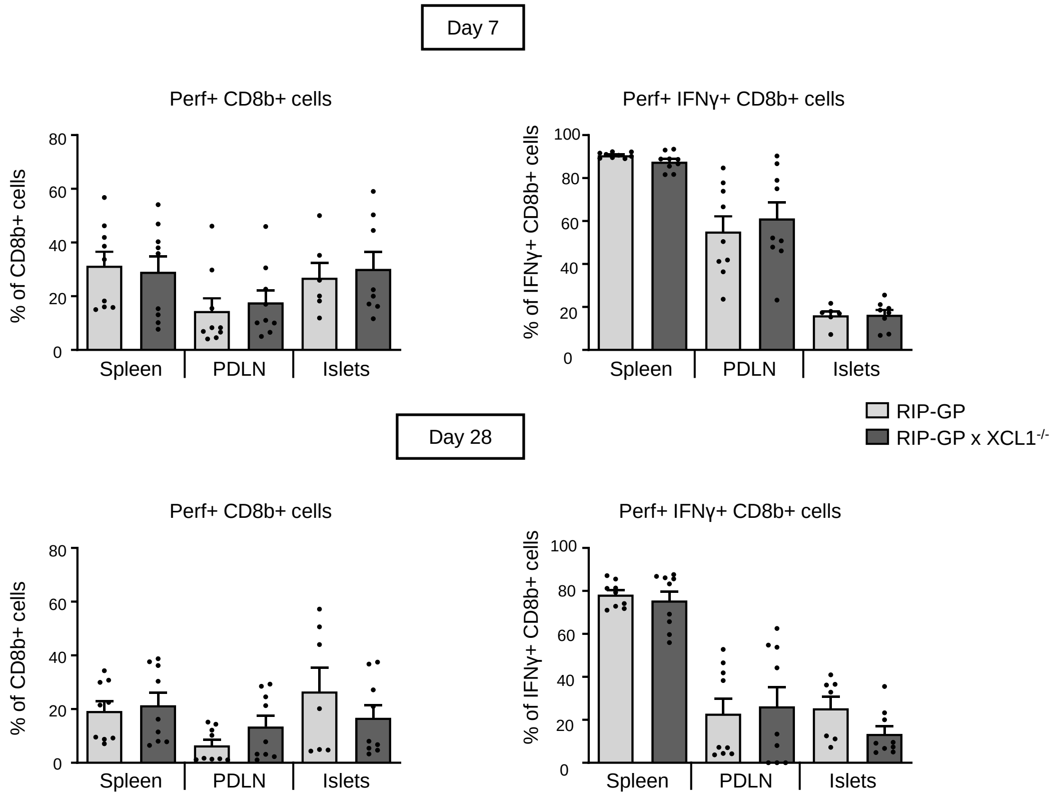

Supplemental Fig.S6 Frequencies of perforin+ (Perf+) cells of CD8b+ cells and perforin+ LCMV-GP33 specific (IFNγ) CD8b+ cells. Frequencies of perforin+ (Perf+) cells of CD8b+ cells and perforin+ LCMV-GP33 specific (IFNγ) CD8b+ cells. in the different organs (spleen, pancreatic draining lymph nodes and islets) obtained via flow cytometric analysis at day 7 and day 28 after infection, comparing RIP-GP mice with RIP-GP x XCL1$^{-/-}$ mice. Values are displayed as mean ± SEM and significant p-values are indicated (n=6-9).
